# Phase I study of dose-escalated paclitaxel, ifosfamide, and cisplatin (PIC) combination chemotherapy in advanced solid tumours

**DOI:** 10.1054/bjoc.1999.0919

**Published:** 2000-01-18

**Authors:** C Kosmas, N B Tsavaris, A Polyzos, N A Malamos, M Katsikas, M J Antonopoulos

**Affiliations:** Department of Medicine, Medical Oncology Unit, Helena-Venizelou Hospital, 21 Apolloniou Street, Athens, 16341, Greece; Medical Oncology Unit, Laikon General Hospital, Athens University School of Medicine, Athens, Greece

**Keywords:** paclitaxel, ifosfamide, cisplatin, phase I study

## Abstract

Based on the already known in vitro synergy between paclitaxel (taxol), cisplatin and oxazophosphorine cytostatics and the broad spectrum of activity of the above drugs we sought to evaluate the paclitaxel (taxol)-ifosfamide-cisplatin (PIC) combination in the outpatient setting in individuals with a variety of advanced solid tumours. Cohorts of patients were entered into six successive dose levels (DLs) with drug doses ranging as follows: paclitaxel 135–215 mg m^−2^day 1 – (1 h infusion), ifosfamide 4.5–6.0 g m^−2^(total dose) – divided over days 1 and 2, and cisplatin 80–100 mg m^−2^(total) – divided over days 1 and 2. Granulocyte colony-stimulating factor was given from day 5 to 14. Forty-two patients were entered. Eighteen patients had 2–8 cycles of prior chemotherapy with no taxanes or ifosfamide (cisplatin was allowed). The regimen was tolerated with outpatient administration in 36/42 patients. Toxicities included: grade 4 neutropenia for ≤ 5 days in 27% of cycles; 5 episodes of febrile neutropenia in three patients at DL-III, -V and -VI. Grade 3/4 thrombocytopenia and cumulative grade 3 anaemia were seen in 7% and 13% of cycles respectively. Three cases of severe grade 3 neuromotor/sensory neuropathy were recorded at DL-II, -III, and -V, all after cycle 3. The maximum tolerated dose was not formally reached at DL-V, but because of progressive anaemia and asthenia/fatigue, it was decided to test a new DL-VI with doses of paclitaxel 200 mg m^−2^, ifosfamide 5.0 g m^−2^and cisplatin 100 mg m^−2^; this appeared to be tolerable and is recommended for further phase II testing. The response rate was 47.5% (complete response + partial response: 20/42). The PIC regimen appears to be feasible and safe in the outpatient setting. Care should be paid to neurotoxicity. Phase II studies are starting in non-small-cell lung cancer, ovarian cancer and head and neck cancer at DL-VI. © 2000 Cancer Research Campaign


*British Journal of Cancer* (2000) **82**(2), 300–307
© 2000 Cancer Research Campaign
Article no. bjoc.1999.0919

### Phase I study of dose-escalated paclitaxel, ifosfamide, and cisplatin (PIC) combination chemotherapy in advanced solid tumours

**C Kosmas[1], NB Tsavaris[2], A Polyzos[2], NA Malamos[1], M Katsikas[2] and MJ Antonopoulos[1]**

[1]Department of Medicine, Medical Oncology Unit, Helena-Venizelou Hospital, 21 Apolloniou Street, 16341 Athens, Greece; [2]Medical Oncology Unit, Laikon General Hospital, Athens University School of Medicine, Athens, Greece

**Summary** Based on the already known in vitro synergy between paclitaxel (taxol), cisplatin and oxazophosphorine cytostatics and the broad spectrum of activity of the above drugs we sought to evaluate the paclitaxel (taxol)-ifosfamide-cisplatin (PIC) combination in the outpatient setting in individuals with a variety of advanced solid tumours. Cohorts of patients were entered into six successive dose levels (DLs) with drug doses ranging as follows: paclitaxel 135–215 mg $m^{-2}$ day 1 – (1 h infusion), ifosfamide 4.5–6.0 g $m^{-2}$ (total dose) – divided over days 1 and 2, and cisplatin 80–100 mg $m^{-2}$ (total) – divided over days 1 and 2. Granulocyte colony-stimulating factor was given from day 5 to 14. Forty-two patients were entered. Eighteen patients had 2–8 cycles of prior chemotherapy with no taxanes or ifosfamide (cisplatin was allowed). The regimen was tolerated with outpatient administration in 36/42 patients. Toxicities included: grade 4 neutropenia for ≤ 5 days in 27% of cycles; 5 episodes of febrile neutropenia in three patients at DL-III, -V and -VI. Grade 3/4 thrombocytopenia and cumulative grade 3 anaemia were seen in 7% and 13% of cycles respectively. Three cases of severe grade 3 neuromotor/sensory neuropathy were recorded at DL-II, -III, and -V, all after cycle 3. The maximum tolerated dose was not formally reached at DL-V, but because of progressive anaemia and asthenia/fatigue, it was decided to test a new DL-VI with doses of paclitaxel 200 mg $m^{-2}$, ifosfamide 5.0 g $m^{-2}$ and cisplatin 100 mg $m^{-2}$; this appeared to be tolerable and is recommended for further phase II testing. The response rate was 47.5% (complete response + partial response: 20/42). The PIC regimen appears to be feasible and safe in the outpatient setting. Care should be paid to neurotoxicity. Phase II studies are starting in non-small-cell lung cancer, ovarian cancer and head and neck cancer at DL-VI. © 2000 Cancer Research Campaign

**Keywords:** paclitaxel; ifosfamide; cisplatin; phase I study

Paclitaxel (Taxol®) represents a recently established active cytotoxic agent against a wide variety of advanced solid tumours including ovarian, breast, lung, head and neck cancers, etc. Both ifosfamide and cisplatin have demonstrated activity as single agents and in combination in a broad range of tumours both in first-line treatment as well as in the setting of relapsed/refractory disease.

The potential synergism of paclitaxel and agents that cause DNA damage was evidenced by the observation that paclitaxel enhanced radiation-induced cell death in human glioblastoma cell lines (Tishler et al, 1992*a*, 1992*b*). In addition, several groups have demonstrated that paclitaxel intensifies the cell-killing effects of chemically-induced DNA damage by alkylating agents and cisplatin, provided that paclitaxel precedes these agents. A supra-additive cell killing effect was observed when certain cell lines were incubated with paclitaxel combined with cisplatin (Parker et al, 1993).

In the clinical setting, paclitaxel has shown enhanced activity and possibly synergistic effects when combined with alkylating agents cyclophosphamide/ifosfamide (Bunnell et al, 1998) or cisplatin (Rowinsky et al, 1993). However, ifosfamide has shown to synergize with platinum compounds by reversing intracellular mechanisms of resistance that would ultimately lead to increased DNA repair and/or detoxification of reactive intermediates of cisplatin, such as the glutathione/thiol (GSH) systems. Depletion of the intracellular glutathione pool by 70% has been observed in peripheral blood lymphocytes after ifosfamide administration (Lind et al, 1989). It is thus theoretically conceivable that the administration of ifosfamide and cisplatin might overcome resistance to cisplatin due to elevated GSH concentrations. Therefore, given the in vitro and in vivo synergism of every selected pair of drugs between paclitaxel, ifosfamide and cisplatin, it is expected that the three-drug combination (given the acronym PIC by our group) would exert a favourable cytotoxic profile against a variety of advanced solid tumours. In addition, the safety of the 1-h paclitaxel infusion schedule given with short premedication, studied by our group and other investigators, made the drug very easy to administer in the out-patient setting, as well as allowed its incorporation into complex multidrug chemotherapy regimens, such as the combination of paclitaxel, ifosfamide and cisplatin (Greco and Hainsworth, 1995; Tsavaris et al, 1997; Tsavaris and Kosmas, 1998).

The aim of the present study was to (i) evaluate the maximum tolerated dose (MTD) of the combination of paclitaxel–ifosfamide–cisplatin (PIC), drugs known to possess significant single-agent and doublet activity against a wide variety of solid tumours, as well as carefully define dose-limiting toxicities (DLT), (ii) obtain preliminary evidence of activity against a variety of advanced solid tumours, (iii) examine the feasibility of out-patient administration of a regimen containing drugs characterized for the

*Received 6 May 1999*
*Revised 26 July 1999*
*Accepted 5 August 1999*

*Correspondence to:* C Kosmas, E-mail: ckosm@ath.forthnet.gr preference to be given for safety in the in-patient setting, due to the requirement of vigorous pre- and post-hydration and electrolyte replacement, and thus reduce hospitalization costs.

### PATIENTS AND METHODS

### Patient selection

Patients with histologically confirmed carcinomas and no curative option with surgery, radiation therapy, or any other chemotherapy regimen were candidates for the present study. Eligibility included: (i) histologically confirmed malignancy not curable by standard chemotherapy; (ii) WHO performance status ≤ 2; (iii) life expectancy ≥ 3 months; (iv) adequate haematopoietic (ANC > 1500 $\mu l^{-1}$, PLT > 100 000 $\mu l^{-1}$), liver (bilirubin < 1.5 mg $dl^{-1}$, AST/ALT < 2 × upper normal limit (nl), unless caused by tumour and serum albumin > 3.0 g $dl^{-1}$) and renal function (BUN and creatinine < 1.5 nl; nl = 1.5 mg $dl^{-1}$ in our laboratory or creatinine clearance > 50 ml $min^{-1}$); (v) no previous chemotherapy earlier than 4 weeks from study entry (6 weeks for nitrosoureas and mitomycin-C); (vi) no prior treatment with taxanes or ifosfamide, while cisplatin or carboplatin pre-treatment was allowed; (vii) absence of active coronary artery disease (in the form of unstable angina or myocardial infarction over the last 12 months), unstable diabetes mellitus, or peripheral neuropathy ≥ grade 2 by the NCI-Common Toxicity Criteria (CTC); (viii) no prior irradiation to areas encompassing > 30% of marrow-bearing bone; and (ix) presence of bi-dimensionally measurable disease outside a previously irradiated field, unless definite evidence of progression at this site. The study was approved by the Institutional Review Board of the participating hospitals.

### Treatment schedule

Eligible patients were entered in the dose levels as shown in Table 1. Paclitaxel (Taxol®) was administered at 135–215 mg $m^{-2}$ over 1 h by intravenous (i.v.) infusion on day 1, after premedication consisting of dexamethasone 20 mg, dimethidene maleate (Fenistil®) 4 mg and ranitidine 50 mg; all administered i.v. 1 h before paclitaxel (8). Ifosfamide was administered at 4.5–6.0 g $m^{-2}$ i.v. over 1 h divided between 2 days (days 1 and 2: 2.25–3.0 g $m^{-2}$ per day) together with mesna uroprotection, 40% of the ifosfamide dose, given i.v. before, at 3 and 6 h after ifosfamide. Cisplatin 80–100 mg $m^{-2}$ i.v. over 30 min divided between 2 days (days 1 and 2: 40–50 mg $m^{-2}$ per day) with adequate vigorous pre- and post-hydration, furosemide and electrolyte replacement; 20 mEq potassium chloride and 8 mEq magnesium sulphate per litre of post-hydration solution (0.9% normal saline (N/S) or 1/2 N/S + 5% dextrose (D5/W)).

### Supportive care

Standard anti-emetic medication included ondansetron 24 mg i.v. 1 h before chemotherapy, at 12 h 8 mg orally on days 1 and 2 and post-chemotherapy 8 mg three times a day orally on days 3–5. Dexamethasone 20 mg i.v. was administered 1 h before chemotherapy (day 1 as taxol premedication as well) on days 1 and 2 and post-chemotherapy 4 mg three times a day or methylprednisolone 16 mg twice a day orally on days 3–5 (Tsavaris et al, 1998). Haematopoietic growth factors included granulocyte colony-stimulating factor (G-CSF; lenograstim) 5 μg $kg^{-1}$ subcutaneously (s.c.) from day 4 until WBC ≥ 10 000 $\mu l^{-1}$ (all except the first three patients at DL-I).

### Dose escalation schedule, DLTs and dose modifications

Dose-limiting toxicity (DLT) was defined as follows: (i) grade 4 neutropenia of > 7 days duration; (ii) any episode of febrile ≥ grade 3 neutropenia; (iii) any episode of grade 4 thrombocytopenia requiring platelet transfusions; (iv) any non-haematologic grade 3 or 4 toxicity excluding nausea/vomiting, musculoskeletal/arthritic pain and alopecia. Treatment was administered up to a maximum of 6 cycles for responding patients or those with disease stabilization, unless unacceptable toxicity was encountered, as defined in the previous section. Patients with progressive disease (PD) were offered palliative treatment or alternative treatment at the discretion of the treating physician.

Cohorts of five patients were entered at the dose levels shown in Table 1. In the case that DLT was encountered (defined below) in 2/5 patients at a certain dose level, three more patients were entered at that particular level and if ≥ 1/3 met the DLT requirements (in total at least 3/8 patients) it was then considered that the DLT level was reached, otherwise accrual to the next higher dose level was undertaken. In the case that three out of the first five patients at a certain level experienced DLT no more patients were accrued at that level and the immediately prior dose level was considered as the MTD.

The following guidelines were applied with respect to dose reductions for toxicity; (i) for neutropenia meeting DLT criteria paclitaxel and ifosfamide doses were reduced by 20% in subsequent cycles and if toxicity reappeared after a total of 40% reduction from the starting dose at a certain level in consecutive cycles treatment was stopped, however, the patient was evaluable for toxicity and response; (ii) for thrombocytopenia meeting DLT criteria, reduction of cisplatin by 20% was applied in addition to paclitaxel and ifosfamide dose reductions as specified for dose-limiting neutropenia; (iii) for ≥ grade 3 mucositis the doses of paclitaxel and ifosfamide were reduced by 20% in subsequent cycles; (iv) for neuropathy ≥ grade 3 treatment was interrupted; (v) for renal toxicity ≥ 3 grade toxicity (serum creatinine elevations > 3 × normal) treatment was withheld until recovery (serum creatinine < 1.8 mg $dl^{-1}$) with cisplatin and ifosfamide administered with more post-hydration, mannitol diuresis and hospitalization in subsequent cycles. If the glomerular filtration rate (GFR) dropped to < 40 ml $min^{-1}$, cisplatin and ifosfamide were omitted in subsequent cycles; (vi) for ≥ grade 3 central nervous system

**Table 1** PIC dose levels

| | Drug doses | | |
|---|---|---|---|
| Dose level | Paclitaxel (mg $m^{-2}$) | Ifosfamide (g $m^{-2}$) | Cisplatin (mg $m^{-2}$) |
| I | 135 | 4.5 | 80 |
| II | 175 | 4.5 | 80 |
| III | 175 | 4.5 | 100 |
| IV | 215 | 4.5 | 100 |
| V | 215 | 6.0 | 100 |
| VI | 200 | 5.0 | 100 |

© 2000 Cancer Research Campaign

(CNS) toxicity (ifosfamide encephalopathy) the dose of ifosfamide was reduced by 20% and more hydration with bicarbonates was anticipated in subsequent cycles. In the case that encephalopathy reappeared then ifosfamide was omitted from subsequent cycles.

In the case that blood counts had not recovered to ANC $\geq 1500\ \mu l^{-1}$ and PLT $\geq 100\,000\ \mu l^{-1}$ on the day of therapy, treatment was withheld until recovery, and after a maximum delay of 2 weeks no further therapy was administered.

### Pretreatment, follow-up studies and response evaluation

Tumour measurements were performed by physical examination and the specific radiological test that documented measurable disease before treatment. Clinical examination, full blood counts, biochemical tests, appropriate serum tumour marker measurements and a chest X-ray were carried out before each cycle of therapy. Blood counts were checked every 3 days after each cycle until recovery. Evaluation of response was performed every 2 cycles of therapy. Patients experiencing toxic death despite objective responses at measurable sites would be categorized as treatment failures. Complete remission (CR) is defined as the disappearance of all signs and symptoms of disease for at least 1 month, with the documented disappearance of all known lesions by physical examination, X-rays, computerized tomography (CT) scans, bone scans and the development of no new lesions. Partial remission (PR) indicates a decrease of 50% or greater (compared with pretreatment measurements) in the sum of the products of the two largest perpendicular diameters of all measurable lesions and no concomitant growth of new lesions for at least 1 month. There could be no deterioration of symptoms or performance status unless secondary to drug toxicity. Stable disease (SD) indicates a decrease of less than 50% or an increase in tumour size less than 25% over the original measurements. There could be no deterioration of symptoms or performance status unless secondary to drug toxicity. Progressive disease (PD) was defined as an increase of 25% or greater over the original measurements in the sum of the products of the two largest perpendicular diameters of any measurable lesions, and relapse was defined as occurring following a period of response when a former lesion reappeared or enlarged as above or a new lesion appeared.

Full-staging evaluation had to be performed, as reported above, before treatment initiation. Follow-up disease evaluation was performed at approximately 3-month intervals after the end of treatment.

### RESULTS

### Patient characteristics

Forty-two patients were entered in the present study. Their pretreatment characteristics are shown in Table 2, with all being evaluable for toxicity and all but one for response. Table 3 demonstrates the DLs studied, the number of patients enroled at each DL, the number of patients requiring dose reductions as a result of toxicity and the number of evaluable courses. Seven patients required 1 or more dose reductions because of toxicity, mainly in the form of myelosuppression; three patients – five dose reductions, peripheral neuropathy (grade 3); three patients – two omissions of the planned last cycle, however, no evidence of ≥ grade 3 neuropathy was encountered before cycle 4, and severe (grade 3) asthenia-fatigue; one patient – omission of the last two cycles of PIC. No dose reductions or schedule modifications were required for renal toxicity. A total of 205 courses were evaluable for toxicity. One patient died after cycle 1 at DL-III from an acute bowel haemorrhage and multiorgan failure despite recovering from grade 4 neutropenia and grade 3 thrombocytopenia on day 16. The death, however, was

**Table 2** Patient characteristics

| | |
|---|---|
| Number | 42 |
| Men/women | 29/13 |
| Median age (range) | 55 (25–72) |
| Median PS (WHO) (range) | 1 (0–2) |
| Tumour type: | |
| NSCLC | 17 |
| Ovarian cancer | 6 |
| H&N cancer | 5 |
| UNPC | 3 |
| UPC | 2 |
| Cervical cancer | 2 |
| Breast cancer | 2 |
| Bladder cancer | 2 |
| GCT | 1 |
| Oesophageal cancer | 1 |
| Anal canal cancer | 1 |
| Prior therapy: | 27 |
| Sx only | 4 |
| Sx + RT | 3 |
| Sx + CT | 7 |
| Sx + RT + CT | 1 |
| RT only | 2 |
| RT + CT | 6 |
| CT only | 4 |
| No of prior CT regimens: | |
| 0 | 24 |
| 1 | 17 |
| 2 | 1 |

NSCLC: non-small-cell lung cancer; H&N: head and neck; UNPC: undifferentiated nasopharyngeal carcinoma; UPC: unknown primary carcinoma; GCT: germ-cell tumour; Sx: surgery; RT: radiotherapy; CT: chemotherapy.

**Table 3** Dose-limiting toxicity (DLT) at each level

| | No. of patients | | | |
|---|---|---|---|---|
| Dose level | Entered | With DLT | Type of DLT | Total no. of cycles (range) |
| I | 5 | 0 | — | 28 (4–6) |
| II | 8 (2)[a] | 2 | FN + PLT, PNs | 44 (3–6) |
| III | 8 (2) | 2 | TD, PNs+m | 38 (1–6) |
| IV | 5 | 0 | — | 23 (3–6) |
| V | 8 (2) | 2 | FN+PNs+m, A-F | 41 (3–6) |
| VI | 8 (1) | 1 | FN+A-F | 37 (2–6) |

[a]Numbers in brackets represent the number of patients that required dose reduction in subsequent cycles as a result of toxicity. One patient at DL-III and one at DL-V, without toxicity necessitating dose reduction, interrupted treatment both after cycle 4 because of progressive decline in PS and grade III asthenia-fatigue respectively. FN: febrile neutropenia, PN: peripheral neuropathy; PNm: peripheral neuropathy motor; PNs: peripheral neuropathy sensory; A-F: asthenia-fatigue; TD: toxic death; '+' denotes more than one DLT experienced in a certain patient.

*© 2000 Cancer Research Campaign*

**Table 4** Haematological toxicity (HT)

| | | Dose Level | | | | | | |
|---|---|---|---|---|---|---|---|---|
| | | **I** | **II** | **III** | **IV** | **V** | **VI** | **Total** |
| **No. of assessable patients** | | **5** | **8** | **8** | **5** | **8** | **8** | **42** |
| Courses assessable for HT (range/patient; median) | | 28 (4–6; 6) | 38 (3–6; 4) | 38 (1–6; 5) | 23 (3–6; 4) | 41 (3–6; 6) | 37 (2–6; 6) | 205 |
| No. of courses with (%): | | | | | | | | |
| Leukopenia | G1 | 6 (21) | 16 (42) | 8 (21) | 8 (35) | 8 (20) | 8 (22) | 54 (26) |
| | G2 | 0 (0) | 7 (18) | 2 (5) | 0 (0) | 1 (2) | 11 (30) | 21 (10) |
| | G3 | 9 (32) | 6 (16) | 10 (26) | 3 (13) | 13 (32) | 9 (24) | 50 (24) |
| | G4 | 0 (0) | 4 (11) | 7 (18) | 4 (17) | 10 (24) | 0 (0) | 25 (12) |
| Neutropenia | G1 | 7 (25) | 7 (18) | 2 (5) | 7 (30) | 2 (5) | 6 (16) | 31 (15) |
| | G2 | 1 (4) | 4 (11) | 4 (11) | 1 (4) | 4 (10) | 7 (19) | 21 (10) |
| | G3 | 7 (25) | 6 (16) | 7 (18) | 5 (22) | 13 (32) | 8 (22) | 46 (22) |
| | G4 | 2 (7) | 10 (26) | 15 (39) | 5 (22) | 13 (32) | 10 (27) | 55 (27) |
| Thrombocytopenia | G1 | 2 (7) | 8 (21) | 13 (32) | 1 (4) | 9 (22) | 14 (38) | 46 (22) |
| | G2 | 9 (32) | 7 (18) | 4 (11) | 3 (13) | 4 (10) | 7 (19) | 7 (19) |
| | G3 | 2 (7) | 1 (3) | 2 (5) | 4 (17) | 4 (10) | 0 (0) | 13 (6) |
| | G4 | 0 (0) | 3 (8) | 0 (0) | 0 (0) | 0 (0) | 0 (0) | 3 (1) |
| Anaemia | G1 | 7 (25) | 11 (29) | 7 (18) | 11 (48) | 20 (49) | 19 (51) | 75 (37) |
| | G2 | 4 (14) | 4 (11) | 8 (21) | 2 (9) | 14 (34) | 5 (14) | 37 (18) |
| | G3/4 | 2 (7) | 7 (18) | 6 (16) | 1 (4) | 7 (17) | 4 (11) | 27 (13) |
| [a]{[Anaemia ≥ G3 (after cycle 4)] | — | 0 (0) | 1 (12.5) | 1 (12.5) | 1 (20) | 3 (37.5) | 1 (12.5) | —} |
| Transfusions of pRBCs | | 1 (4) | 0 (0) | 4 (11) | 0 (0) | 4 (10) | 2 (5) | 11 (5) |
| Febrile neutropenia | | 0 (0) | 2 (5) | 0 (0) | 0 (0) | 2 (5) | 1 (3) | 5 (2.5) |

pRBCs: packed red blood cells; G1–4: Grades 1–4.
[a]Denotes number of patients with ≥ G3 anaemia after cycle 4 (cumulative anaemia).

considered to be treatment-related, although large ascites and intra-abdominal metastases were present; no post-mortem evaluation was carried out. Eighteen patients did not complete the planned cycles of therapy because of: other treatment (radiotherapy), four patients (one each at DL-I, -III, -IV and -VI); toxicity, three patients (one at DL-II and two at DL-V); toxic death, one patient (at DL-III); refusal for personal reasons, four patients (one each at DL-II, -IV, -V and -VI); PD, five patients (two at DL-II, and one each at DL-III, -IV and -VI); and declining PS, one patient at DL-II.

### Haematological toxicity

Haematological toxicities are represented in Table 4. Grade 3 and 4 neutropenia occurred at all DLs (except for grade 4 neutropenia in DL-I) in 49% of treatment courses. However, grade 4 neutropenia whenever encountered did not exceed 5 days, thus, never meeting the definition of DLT requirements (> 7 days' duration) in the absence of fever. G-CSF was administered in all patients except the first three at DL-I in cycles 1/2 only and, it was soon realized, as a result of grade 3 neutropenia, that substantial dose escalation at higher DLs could not be envisaged without G-CSF support. A total of five courses (2.5%) in three patients were associated with febrile neutropenia at DL-II, -V and -VI. The neutrophil nadir was consistently observed between days 8 and 12. Grade 3/4 thrombocytopenia was rare (7% of courses) and in only one occasion PLT transfusions were required (PLT < 5000 $\mu l^{-1}$ without bleeding). Severe anaemia (grade 3/4) was observed during 13% of courses with 5% requiring transfusions of packed red cells.

### Non-haematological toxicities

Non-haematological toxicities are shown in detail in Table 5. The major non-haematological toxicity encountered was a peripheral sensorimotor neuropathy. It was rarely severe; grade 3 in 7% of patients and no case of grade 4, but commonly grade 2 (55%). Peripheral neuropathy tended to be cumulative in nature and observed over the last three cycles, with one patient experiencing grade 3 neuropathy after the last treatment course, while another with a relapsed GCT treated at DL-V (prior exposure to a dose intensive cisplatin/vincristine regimen and high cumulative cisplatin dose) developed it soon after cycle 3, but recovered to grade 2 after 1 month. Most patients, including those with grade 2 neuropathy, found this the most troublesome of all effects. Other toxicities of less importance included: very mild mucositis, 8% grade 2 and 0% grade 3/4 (particularly in head and neck (H&N) cancer patients with prior irradiation to these sites); controllable nausea and vomiting, 9% grade 3–0% grade 4, diarrhoea, 13% grade 1/2 only; orthostatic hypotension, 7% grade 1/2 only; moderate hypomagnesemia, 10% grade 1/2 only; fully reversible renal toxicity (creatinine elevations), 3.5% grade 1/2 only. CNS toxicity related to ifosfamide was mainly grade 1 (23% of courses), very rarely grade 2 (1%), and in one course (0.5%) reached grade 3 in a patient with GCT who developed concurrently a grade 2 creatinine elevation and grade 3 peripheral neuropathy after cycle 3, that was fully reversible. Myalgias and arthralgia potentially attributable to either G-CSF or paclitaxel were of grade 1/2 severity in 59% of cycles, starting at least 48 h after paclitaxel and lasting up to 3–4 days, but rarely posed a significant problem apart from patient anxiety. Alopecia was very common (86% of patients) and occurred at all DLs. Skin toxicity

© 2000 Cancer Research Campaign

**Table 5** Non-haematological toxicity (NHT)

| | | Dose Level | | | | | | |
|---|---|---|---|---|---|---|---|---|
| | | I | II | III | IV | V | VI | Total |
| **No. of assessable patients** | | 5 | 8 | 8 | 5 | 8 | 8 | 42 |
| Courses assessable for NHT | | 28 | 38 | 38 | 23 | 41 | 37 | 205 |
| (range/patient; median) | | (4–6; 6) | (3–6; 4) | (1–6; 5) | (3–6; 4) | (3–6; 6) | (2–6; 6) | |
| No. of courses with (%): | | | | | | | | |
| Nausea & vomiting | G1 | 12 (43) | 20 (53) | 23 (61) | 4 (17) | 8 (20) | 18 (49) | 72 (37) |
| | G2 | 2 (7) | 0 (0) | 8 (21) | 3 (13) | 6 (15) | 4 (11) | 23 (11) |
| | G3 | 1 (4) | 0 (0) | 2 (5) | 6 (26) | 6 (15) | 4 (11) | 19 (9) |
| | G4 | 0 (0) | 0 (0) | 0 (0) | 0 (0) | 0 (0) | 0 (0) | 0 (0) |
| Mucositis | G1 | 9 (32) | 20 (53) | 23 (61) | 4 (17) | 5 (12) | 14 (38) | 75 (37) |
| | G2 | 3 (11) | 1 (3) | 5 (13) | 6 (26) | 1 (2) | 0 (0) | 16 (8) |
| | G3/4 | 0 (0) | 0 (0) | 0 (0) | 0 (0) | 0 (0) | 0 (0) | 0 (0) |
| Diarrhoea | G1 | 1 (4) | 10 (26) | 6 (16) | 0 (0) | 4 (10) | 4 (11) | 25 (12) |
| | G2 | 0 (0) | 0 (0) | 0 (0) | 0 (0) | 2 (5) | 0 (0) | 2 (1) |
| | G3/4 | 0 (0) | 0 (0) | 0 (0) | 0 (0) | 0 (0) | 0 (0) | 0 (0) |
| Myalgia/arthralgia | G1 | 10 (36) | 18 (47) | 10 (26) | 13 (57) | 16 (39) | 21 (57) | 88 (43) |
| | G2 | 0 (0) | 0 (0) | 15 (39) | 0 (0) | 3 (7) | 14 (38) | 32 (16) |
| | G3/4 | 0 (0) | 0 (0) | 0 (0) | 0 (0) | 0 (0) | 0 (0) | 0 (0) |
| Orthostatic hypotension | G1 | 1 (4) | 3 (8) | 1 (3) | 2 (9) | 1 (2) | 3 (8) | 11 (5) |
| | G2 | 0 (0) | 0 (0) | 2 (5) | 0 (0) | 1 (2) | 1 (3) | 5 (2) |
| | G3 | 0 (0) | 0 (0) | 1 (3) | 0 (0) | 0 (0) | 0 (0) | 1 (0.5) |
| | G4 | 0 (0) | 0 (0) | 0 (0) | 0 (0) | 0 (0) | 0 (0) | 0 (0) |
| Hypomagnesemia | G1 | 1 (4) | 3 (8) | 7 (18) | 2 (9) | 3 (7) | 1 (3) | 17 (8) |
| | G2 | 0 (0) | 1 (3) | 1 (3) | 1 (4) | 1 (2) | 0 (0) | 4 (2) |
| | G3/4 | 0 (0) | 0 (0) | 0 (0) | 0 (0) | 0 (0) | 0 (0) | 0 (0) |
| CNS toxicity | G1 | 12 (43) | 9 (24) | 8 (21) | 2 (9) | 5 (12) | 12 (32) | 48 (23) |
| | G2 | 0 (0) | 1 93) | 1 (3) | 0 (0) | 1 (2) | 0 (0) | 2 (1) |
| | G3 | 0 (0) | 0 (0) | 0 (0) | 0 (0) | 1 (2) | 0 (0) | 1 (0.5) |
| | G4 | 0 (0) | 0 (0) | 0 (0) | 0 (0) | 0 (0) | 0 (0) | 0 (0) |
| Renal | G1 | 0 (0) | 0 (0) | 4 (11) | 0 (0) | 0 (0) | 0 (0) | 4 (2) |
| | G2 | 0 (0) | 0 (0) | 1 (3) | 1 (4) | 1 (2.5) | 0 (0) | 3 (1.5) |
| | G3/4 | 0 (0) | 0 (0) | 0 (0) | 0 (0) | 0 (0) | 0 (0) | 0 (0) |
| No. of patients with (%): | | | | | | | | |
| Peripheral neuropathy | G1 | 3 (60) | 1 (12.5) | 2 (25) | 1 (20) | 4 (50) | 2 (25) | 13 (31) |
| | G2 | 2 (40) | 6 (75) | 3 (37.5) | 4 (80) | 3 (37.5) | 5 (62.5) | 23 (55) |
| | G3 | 0 (0) | 1 (12.5) | 1 (12.5) | 0 (0) | 1 (12.5) | 0 (0) | 3 (7) |
| | G4 | 0 (0) | 0 (0) | 0 (0) | 0 (0) | 0 (0) | 0 (0) | 0 (0) |
| Asthenia/fatigue | G1 | 2 (40) | 4 (50) | 3 (37.5) | 4 (80) | 1 (12.5) | 2 (25) | 12 (29) |
| | G2 | 2 (40) | 2 (25) | 2 (25) | 1 (20) | 4 (50) | 4 (50) | 15 (36) |
| | G3 | 0 (0) | 1 (12.5) | 0 (0) | 0 (0) | 3 (37.5) | 0 (0) | 4 (10) |
| Alopecia | G1 | 0 (0) | 0 (0) | 0 (0) | 0 (0) | 0 (0) | 0 (0) | 0 (0) |
| | G2 | 0 (0) | 1 (12.5) | 2 (25) | 0 (0) | 1 (12.5) | 2 (25) | 6 (14) |
| | G3 | 5 (100) | 7 (87.5) | 6 (75) | 5 (100) | 7 (87.5) | 6 (75) | 36 (86) |
| Skin toxicity | G1 | 2 (40) | 3 (37.5) | 4 (50) | 1 (20) | 2 (25) | 3 (37.5) | 15 (36) |
| | G2 | 1 (20) | 1 (12.5) | 0 (0) | 1 (20) | 0 (0) | 0 (0) | 3 (7) |
| | G3 | 0 (0) | 0 (0) | 0 (0) | 0 (0) | 0 (0) | 0 (0) | 0 (0) |

was mild and consisted of increased thickening and facial folding, while no eruptions or rashes and discoloration were observed. A significant toxicity reported by most patients was asthenia/fatigue, occurring in the majority of patients (grade 1 = 45%, grade 2 = 33%, grade 3 = 7%) at almost all DLs, being cumulative in nature, particularly after cycle 4. Paclitaxel-related hypersensitivity reactions were observed in two cases and consisted of very mild facial flushing of brief duration that did not necessitate drug discontinuation.

### Responses

Major responses (CR/PR) were seen in 47.5% of patients, particularly those with NSCLC; 53%, OC; 50%, H&N cancer (with UNPC); 65% (Table 6). It should be noted that many patients were pretreated by chemotherapy and/or radiotherapy (RT) (Table 2). Two NSCLC patients with brain metastases were treated with PIC before cranial RT and had a near CR. All but one ovarian cancer (OC) patient were pretreated with carboplatin ± cyclophosphamide, while 4/5 H&N and 2/3 UNPC patients had prior chemo-RT.

### DISCUSSION

The rationale for combining paclitaxel, ifosfamide and cisplatin derives from both in vitro data and theoretical assumptions based on the properties of each individual cytotoxic agent to mediate its cellular damage. In brief, paclitaxel inhibits the energy-dependent enzymatic reactions, by disengaging activated intracellular phosphate (e.g. ATP and GTP), required for the repair of the DNA

 © 2000 Cancer Research Campaign

**Table 6** Response to treatment; total and by tumour type

| Tumour type | No of patients | Response to treatment (PIC) No of patients (%) | | | |
|---|---|---|---|---|---|
| | | CR | PR | SD | PD |
| NSCLC | 17 | 1 (6) | 8 (47) | 6 (35) | 2 (12) |
| Ovarian cancer | 6 | 2 (33) | 1 (17) | 2 (33) | 1 (17) |
| H&N cancer | 5 | – (0) | 3 (60) | 2 (40) | – (0) |
| UNPC | 3 | – (0) | 2 (67) | 1 (33) | – (0) |
| UPC | 2 | – (0) | – (0) | 1 (50) | 1 (50) |
| Cervical cancer | 2 | – (0) | – (0) | 2 (100) | – (0) |
| Breast cancer | 2 | – (0) | 1 (50) | – (0) | 1 (50) |
| Bladder cancer | 2 | – (0) | 1 (50) | 1 (50) | – (0) |
| GCT | 1 | – (0) | 1 (100) | – (0) | – (0) |
| Oesophageal cancer | 1 | – (0) | – (0) | 1 (100) | – (0) |
| Anal canal cancer | 1 | – (0) | – (0) | 1 (100) | – (0) |
| Total | 42 | 3 (7) | 17 (40.5) | 17 (40.5) | 5 (12) |

damage induced by cisplatin (causing kinking of the DNA double helix) and oxazaphosphorine (cyclophosphamide and ifosfamide) alkylating agents (prevention of DNA strand preparation and unwinding). These different types of DNA lesion caused by cisplatin and oxazaphosphorine cytostatics are repaired by the nucleotide excision repair pathway (ERCC and XP genes) and the mismatch repair pathway (*HNPCC* gene) (Reed et al, 1995). In vitro synergism has been demonstrated between paclitaxel and hydroperoxy-ifosfamide, an activated ifosfamide metabolite, against cisplatin-sensitive and -resistant OC cell lines (Klaassen et al, 1996). This synergism appears to be sequence-dependent and exerted when paclitaxel preceded hydroperoxy-ifosfamide or when exposure to these drugs was simultaneous. In contrast, when exposure to hydroperoxy-ifosfamide preceded that of paclitaxel, clear in vitro antagonism was demonstrated, a finding confirmed with other alkylating agents (Kennedy et al, 1994; Liebmann et al, 1994). As discussed earlier, the synergistic interaction between paclitaxel and DNA-damaging agents is based on the ability of paclitaxel to slow the DNA repair processes. This might explain the importance of administering paclitaxel before the DNA-damaging agent.

Based on these preclinical in vitro experimental data, we believe that the sequence and infusion times regarding paclitaxel, ifosfamide and cisplatin, as applied in the present study, might lead to potential in vivo synergism between the three drugs. Ifosfamide and cisplatin given alone on day 2 of our treatment schedule should still be modulated by paclitaxel's sustained activity given only on day 1 by 1 h short infusion. After short 1- and 3-h paclitaxel infusion schedules biologically relevant concentrations ($\geq 0.1\ \mu mol\ l^{-1}$) of the drug are still present at 24-h post-infusion and are rather adequate in inducing pertinent antimicrotubule effects (Kearns et al, 1995). Therefore, synergism between paclitaxel and ifosfamide-cisplatin should apparently be effected over both days of our treatment schedule.

If the above considerations regarding sequence-dependent interactions for optimal drug scheduling are important in order to maximize efficacy, of equal importance are the effects of drug sequencing related to bone marrow toxicity. Data from phase I clinical studies of the paclitaxel/cyclophosphamide combination employing different schedules of drug administration demonstrated variable haematologic toxicity. The highest degree of haematologic toxicity was encountered when paclitaxel was administered by 24-h or 72-h continuous infusion with high doses of cyclophosphamide (Kennedy et al, 1994; Tolcher, 1996). However, when paclitaxel, given by 3-h infusion, was followed by cyclophosphamide, bone marrow toxicity was of much less severity (Pagani et al, 1997). Toxicity with the paclitaxel/cyclophosphamide combination appears to be lessened when paclitaxel follows cyclophosphamide. Similarly, with the docetaxel/ifosfamide combination, the sequence of giving the taxane first led to a higher MTD than the reverse (Pronk et al, 1998). However, sequence dependence may be less apparent with concurrent or near-concurrent administration of paclitaxel and cyclophosphamide, as it appears to be the case with the short paclitaxel infusion schedules (Pagani et al, 1997). It is therefore realistic to consider that the almost concurrent administration of paclitaxel and ifosfamide followed by cisplatin could account for the tolerable haematologic toxicity, i.e. neutropenia and thrombocytopenia, encountered in our study up to high individual drug doses given at DL-V. At the highest DL, such doses of ifosfamide ($6\ g\ m^{-2}$) and cisplatin ($100\ mg\ m^{-2}$) when combined with other myelotoxic drugs, like etoposide, are associated with a high incidence of thrombocytopenia. The very low incidence of grade 4 platelet toxicity even at these doses combined with paclitaxel $215\ mg\ m^{-2}$ might imply a megakaryocyte or marrow progenitor cell sparing effect exerted by paclitaxel, closely similar to the situation postulated to occur when paclitaxel is combined with carboplatin, a classic platelet cytotoxin (Huizing et al, 1997).

Neurotoxicity, in the form of peripheral neuropathy, is anticipated to be the principal non-haematologic toxicity when evaluating paclitaxel–cisplatin combinations. In our study, only three cases of grade 3 peripheral neuropathy were recorded, one each at DL-II, DL-III and DL-V, principally consisting of severe dysaesthesias in two patients, loss of proprioception in one patient, while all patients developed motor dysfunction as well as numbness, burning and parasthesias. It should be noted that 2/3 of these patients were pre-treated with potent neurotoxic drugs; a woman with a relapsed cervical carcinoma had received prior chemotherapy with a weekly combination of bleomycin, vincristine and cisplatin (BOP regimen) × 4 weeks and a man with relapsed GCT had been pretreated with the intensive BOP-BEP regimen for high-risk GCTs (Horwich et al, 1994), both incorporating aggressive cisplatin and vincristine dosing. Peripheral neuropathy appeared to be cumulative in nature, usually occurring

*© 2000 Cancer Research Campaign*

after cycle 4 or after the end of the planned six courses. Of the above three patients, who developed severe neuropathy, one did so after cycle 4 (DL-II), one after cycle 6 (DL-III) and the patient with GCT treated at the highest DL (DL-V) after cycle 3, most likely due to his prior exposure to multiple cycles of high-dose cisplatin and vincristine (BOP-BEP regimen). These are close to the toxicity levels achieved with the paclitaxel/cisplatin combination at 250/75 mg $m^{-2}$ (Rowinsky et al, 1993). In this landmark analysis of neurotoxicity, regarding the paclitaxel/cisplatin combination, it became apparent that peripheral neuropathy was cumulative in nature and evident after 4–6 cycles, but at the highest paclitaxel dose levels (≥ 250 mg $m^{-2}$) the onset of toxicity was usually abrupt and appeared relatively early (after 1–2 cycles). Cisplatin appears to aggrevate the neuropathy caused by paclitaxel, since combinations of carboplatin and paclitaxel have shown a lower degree of peripheral neuropathy compared to paclitaxel/cisplatin. Moreover, cisplatin on its own has demonstrated a dose-dependent neurotoxicity profile with most episodes occurring at doses ≥ 100 mg $m^{-2}$. It is therefore possible in our study, combining cisplatin 100 mg $m^{-2}$ (at ≥ DL-III), to produce significant peripheral neuropathy even at lower paclitaxel doses than the ones leading to toxicity when combined with cisplatin 75 mg $m^{-2}$ (Rowinsky et al, 1993). However, as peripheral neuropathy appeared late during or after the entire treatment course, it did not constitute a formal dose-limiting factor, precluding further dose escalation in our phase I study. Given that certain patients did not complete the full treatment course for reasons other than peripheral neuropathy (other therapeutic modality, disease progression, etc.), the incidence of neurotoxicity may have been underestimated in the present study. We think that care should be given with the current combination to patients with prior exposure to neurotoxic drugs, elderly persons (> 60 years), and those with a history of diabetes, chronic alcoholism and medical disorders associated with peripheral neuropathy. Therefore, it would probably be prudent to use lower drug doses when treating patients at risk for developing substantial neurotoxicity.

Despite the absence of dose-limiting neutropenia and/or thrombocytopenia at DL-V precluding further dose escalation, it became apparent that we were reaching the limits of the regimen given the occurrence of cumulative anaemia and asthenia, usually after cycle 4, at this DL. Therefore, we did not feel it realistic to attempt further dose escalation. A more reasonable DL for further phase II testing in non-pretreated patients would be DL-VI, which does not result in significant toxicities or asthenia with decline in PS.

Preliminary results of the current combination with either cisplatin or carboplatin have been reported by various investigators (Palackdhary, 1997; Zaniboni et al, 1997; Bajorin et al, 1998; Shin et al, 1998; Zanetta et al, 1998). All but one study have not attempted defining DLTs and have chosen arbitrarily doses of drugs much lower than those reached in our study (Palackdhary, 1997). In that study, where a formal phase I design was undertaken, only very preliminary results in ten patients have been presented.

Despite the fact that tumour response was not the primary objective of the present study a 47.5% response rate was encountered (Table 6), with many responding patients having had failed prior chemotherapy ± RT. Responses were seen in NSCLC, OC and H&N cancer patients. Similarly, high response rates have been observed by other investigators in H&N cancer (Shin et al, 1998), bladder cancer (Bajorin et al, 1998), cervical cancer (Zanetta et al, 1998), NSCLC (Zaniboni et al, 1997) and GCTs (Motzer et al, 1997). A preliminary phase I/II study of docetaxel–ifosfamide–cisplatin has yielded encouraging results in NSCLC (Donnellan and Crown, 1997).

In conclusion, the current phase I trial of PIC combination has demonstrated the feasibility of the regimen in the out-patient setting, at high individual drug doses, and a promising preliminary activity profile against a variety of advanced solid tumours.

### ACKNOWLEDGEMENT

We are indebted to Mrs Sofia Rokana (BSc in Mathematics) for her assistance in data managing and computing.

### REFERENCES

Bajorin DF, McCaffrey JA, Hilton S, Mazumdar M, Kelly WK, Scher HI, Spicer J, Herr H and Higgins G (1998) Treatment of patients with transitional-cell carcinoma of the urothelial tract with ifosfamide, paclitaxel, and cisplatin: a phase II trial. *J Clin Oncol* **16:** 2722–2727

Bunnell CA, Thompson L, Buswell L, Berkowitz R, Muto M, Sheets E and Shulman LN (1998) A phase I trial of ifosfamide and paclitaxel with granulocyte-colony stimulating factor in the treatment of patients with refractory solid tumors. *Cancer* **82:** 561–566

Donnellan PP and Crown JP (1997) The development of docetaxel (taxotere) in non-small cell lung cancer. Docetaxel in new combinations and new schedules: an overview of ongoing and future developments. *Semin Oncol* **24:** 18–21

Greco FA and Hainsworth JD (1995) One hour paclitaxel infusion schedule: a phase I/II comparative trial. *Semin Oncol* **22:** 118–123

Horwich A, Dearnaley DP, Norman A, Nicolls J and Hendry WF (1994) Accelerated chemotherapy for poor prognosis germ cell tumours. *Eur J Cancer* **30A:** 1607–1611

Huizing MT, van Warmerdarm JC, Rosing H, Schaefers MCW, Lai A, Helmerhorst TJM, Veenhof CHN, Birkhofer MJ, Rodenhuis S, Beijnen JH and ten Bokkel Huinik WW (1997) Phase I and pharmacologic study of the combination paclitaxel and carboplatin as first-line chemotherapy in stage III and IV ovarian cancer. *J Clin Oncol* **15**: 1953–1964

Kearns CM, Gianni L and Egorin MJ (1995) Paclitaxel pharmacokinetics and pharmacodynamics. *Semin Oncol* **22**: 16–23

Kennedy MJ, Armstrong D, Donehower R, Noe D, Sartorius S, Chen T-L, Bowling K and Rowinsky E (1994) The hematologic toxicity of the Taxol/Cytoxan doublet is sequence-dependent. *Proc Am Soc Clin Oncol* **13**: 74 (abstract 342)

Klaassen U, Harstrick A, Schleucher N, Vanhoefer U, Schroder J, Wilke H and Seeber S (1996) Activity and schedule-dependent interactions of paclitaxel, etoposide and hydroperoxy-ifosfamide in cisplatin-sensitive and -refractory human ovarian carcinoma cell lines. *Br J Cancer* **74**: 224–228

Liebmann JE, Fisher J and Teague D (1994) Sequence dependence of paclitaxel (Taxol) combined with cisplatin or alkylators in human cancer cells. *Oncol Res* **6**: 25–31

Lind MJ, McGowan AT, Hadfield JA, Thatcher N, Crowther D and Fox BW (1989) The effect of ifosfamide and its metabolites on intracellular glutathione levels in vitro and in vivo. *Biochem Pharmacol* **38**: 1835–1840

Motzer RJ, Bajorin DF, Bosl GJ, Reich L, Tong W and Green GA (1997) Paclitaxel containing first-line salvage therapy selected by risk for patients with germ cell tumors. *Proc Am Soc Clin Oncol* **13**: 322 (abstract 1146)

Pagani O, Sessa C, Martinelli G, Cerny T, de Jong J, Goldhirsh A, Zimatore M and Cavalli F (1997) Dose-finding study of paclitaxel and cyclophosphamide in advanced breast cancer. *Ann Oncol* **8**: 655–661

Palackdharry CS (1997) Phase I trial of dose-escalated paclitaxel and carboplatin in combination with ifosfamide and filgrastim: preliminary results. *Semin Oncol* **24**: 108–112

Parker RJ, Dabholkar MD, Lee KB, Bostick-Bruton F and Reed E (1993) Taxol effect on cisplatin sensitivity and cisplatin cellular accumulation in human ovarian cancer cells. *J Natl Cancer Inst Monogr* **15**: 83–88

Pronk LC, Schrijvers D, Schellens JHM, de Bruijn EA, Planting ASTh, Locci-Tonelli D, Grouit V, Verweij J and van Oosterom AT (1998) Phase I study of docetaxel and ifosfamide in patients with advanced solid tumors. *Br J Cancer* **77**: 153–158

Reed E, Kohn EC, Sarosy G, Dabholkar M, Davis P, Jacob J and Maher M (1995) Paclitaxel, cisplatin, and cyclophosphamide in human ovarian cancer: molecular rationale and early clinical results. *Semin Oncol* **22**: 90–96

 © 2000 Cancer Research Campaign

Rowinsky EK, Chaudhry V, Forastiere AA, Sartorius SE, Ettinger DS, Grochow LB, Lubejko BG, Cornblath DR and Donehower RC (1993) Phase I and pharmacologic study of paclitaxel and cisplatin with granulocyte colony-stimulating factor: neuromuscular toxicity is dose-limiting. *J Clin Oncol* **11**: 2010–2020

Shin DM, Glisson BS, Khuri FR, Ginsberg L, Papadimitrakopoulou V, Lee JJ, Lawhorn K, Gillenwater AM, Ang K-K, Clayman GL, Callender DL, Hong WK and Lippman SM (1998) Phase II trial of paclitaxel, ifosfamide, and cisplatin in patients with recurrent head and neck squamous cell carcinoma. *J Clin Oncol* **16**: 1325–1330

Tishler RB, Geard CR, Hall EJ and Schiff PB (1992*a*) Taxol sensitizes human astrocytoma cells to radiation. *Cancer Res* **52**: 3495–3497

Tishler RB, Schiff PB, Geard CR and Hall EJ (1992*b*) Taxol: a novel radiation sensitizer. *Int J Rad Oncol Biol Phys* **22**: 613–617

Tolcher AW (1996) Paclitaxel couplets with cyclophosphamide or cisplatin in metastatic breast cancer. *Semin Oncol* **23**: 37–43

Tsavaris N and Kosmas C (1998) Risk of severe acute hypersensitivity reactions after rapid (less than 1-hour) paclitaxel infusion. *Cancer Chemother Pharmacol* **42**: 509–511

Tsavaris N, Polyzos A, Kosmas C, Giannikos L and Gogas J (1997) A feasibility study of one-hour paclitaxel infusion in solid tumors. *Cancer Chemother Pharmacol* **40**: 353–357

Tsavaris N, Fountzilas G, Mylonakis N, Athanassiadis A, Kosmas C, Karakousis C, Bacoyiannis Ch and Kosmidis P (1998) A randomized comparative study of antiemetic prophylaxis with ondansetron in a single 32 mg loading dose vs 8 mg every six hours, in patients under cisplatin-based chemotherapy. *Oncology* **55**: 513–516

Zanetta G, Lissoni A, Pellegrino A, Sessa C, Colombo N, Gueli-Alletti D and Mangioni C (1998) Neoadjuvant chemotherapy with cisplatin, ifosfamide and paclitaxel for locally advanced squamous-cell cervical cancer. *Ann Oncol* **9**: 977–980

Zaniboni A, Meriggi F, Rizzi A, Alghisi A, Pascarella A, Bozzola G, Mutti S and Marini G (1997) Paclitaxel, ifosfamide, and carboplatin for the treatment of stages IIIB and IV non-small cell lung cancer. *Semin Oncol* **24**: 70–72

*© 2000 Cancer Research Campaign*
